# The correlation between renal transplantation and liver carcinoma: a meta-analysis

**DOI:** 10.18632/oncotarget.19456

**Published:** 2017-07-22

**Authors:** Hong Yongzhi, Xu Min, Yu Bo, Chen Pin, Shi Xueqiang

**Affiliations:** ^1^ Department of Neurosurgery, The First Affiliated Hospital of Nanjing Medical University, Nanjing 210029, Jiangsu Province, China; ^2^ Department of Neurosurgery, Kunshan Hospital of Traditional Chinese Medicine, Kunshan Affiliated Hospital of Nanjing University of Chinese Medicine, Kunshan 215300, Jiangsu Province, China; ^3^ Department of Neurosurgery, Clinical Medical College of Yangzhou University, Subei People’s Hospital of Jiangsu province, Yangzhou 225001, Jiangsu Province, China

**Keywords:** meta-analysis, renal transplantation, liver carcinoma, chronic virus hepatitis, anti-virus

## Abstract

**Objective:**

Much of the related researches have reported the correlation between renal transplantation and different tumors in the post transplant recipients. However, there are not exact essays revealed that renal transplantation is definite causation for liver carcinoma, thus we systematically evaluated the association between renal transplantation and the risk of liver carcinoma in this meta-analysis from all available researches.

**Methods:**

All useful data were collected through searching of PubMed and Web of Science until the date of 31 September 2015. Random-effects model were adopted to calculate the standardized incidence ratio and 95% confidence interval (CIs) of the risk of liver carcinoma among renal transplant recipients. Other statistical analyses like heterogeneity tests, sensitivity analysis and publication bias were also performed in this meta-analysis.

**Results:**

Among 17,4256 kidney transplant cases and 25,6736 patients-years observation, 9136 post-transplant cancers were diagnosed. We identified a 2.08-fold higher standardized incidence rate (SIR) (95% confidence interval (CI): 1.24-3.47, *P*=0.005) of liver carcinoma following renal transplantation compared with the general population. Observation and publication bias were not observed in this study.

**Conclusion:**

This study suggested that the risk of liver carcinoma among renal transplant recipients with chronic hepatic disease is higher than general population. Such results alert clinical doctors the importance of anti-virus therapy with chronic virus hepatitis and enough attention of periodic liver screening with chronic liver diseases in renal transplant recipients.

## INTRODUCTION

Chronic kidney disease (CKD) is quite a common disease around the world, its etiology from infectious disease to emerging diseases especially hypertension and diabetes [1–3]. CKD increases a large burden for our society for its highly associated morbidity and mortality, mainly elevated cardiovascular system diseases [4, 5]. In clinical therapy, there are different related therapy ways during CKD patients’ different period, the most common therapy ways like dialysis and renal transplantation.

Renal transplantation is regarded as the therapeutic option of choice in the end-stage renal failure [6]. Initially, for transplant recipients, the *de novo* malignancy is the only sideline of this population, since tumor-specific mortality is not the main reason of the population [7]. However, with the judicious use of better immunosuppressive agents, it dramatically decreases the incidence of acute graft rejection and long-term outcome can be enhanced [8, 9]. At the same time, it increases the risk of variety of new malignancies. The most common of these malignancies are skin cancers and lymphomas, followed by Kaposi, sarcoma, lip, cervical, perineal, renal, hepatocellular carcinomas and other sarcomas [10, 11].

Hepatocellular carcinoma (HCC) is also a common tumor worldwide, it’s popularity has related geographic differences. Epidemiology revealed that areas with high incidence include Africa, in Asia especially southeastern countries (Korea, Hong Kong, Thailand), however, in majority areas of China and Japan. Its main risk factors are hepatitis virus infection like hepatitis B and hepatitis C, the other susceptible factors are alcohol consumption, aflatoxin B1 intake and other associated congenital diseases [12].

Some studies have showed the increased risk of liver carcinoma after renal transplantation [13, 14]. However, not all studies showed similar association around the world, it may relate to the geography and population difference. Because of differences in study design, sample selection, sample size, follow-up period, and population and geographic differences, the connection between renal transplantation and liver carcinoma remains unclear. So this meta-analysis, which included all relevant studies on liver carcinoma and renal transplantation, was performed to clarify whether the total standardized incidence rate (SIR) of liver carcinoma is higher following renal transplantation than in the general population, which might be helpful in determining whether conclusive recommendations for liver cancer screening in renal transplantation recipients are needed.

## RESULTS

### Study characteristics

**Figure 1:**
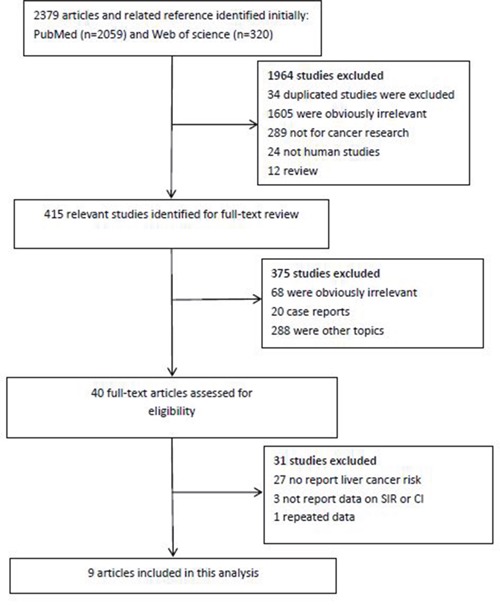
Flow chart of literature search and study selection

A total of 9 studies met our inclusion criteria [13, 15–22]. These 9 articles, which included 17,4256 renal transplantation recipients in 25,7206 patients, evaluated whether the total SIR for liver cancer was higher in renal transplant recipients than in the general population. An outline for the selection process of identified studies is presented in Figure 1. The main characteristics of the studies and their details are listed in Table 1. All the studies were retrospective, and the largest study had 12,0654 renal transplant recipients. These related studies were based on patients in several countries, including Italy, China, Sweden, the USA, the UK, Canada and Japan.

**Table 1:** Summary of studies included in the analysis

Study	Year	Type of transplant	Data source	Geographicorigin	Number of patients (n)	Number of kidneytransplant cases
**Piselli**	2013	Kidney	Italian KT centre	Italy	7217	7299
**Cheung**	2012	Kidney	Hong Kong Renal Registry	China	4674	4895
**Krynitz**	2012	multiorgan	Swedish National Patient Register	Sweden	10476	7952
**Li**	2012	Kidney	Taiwan National Health InsuranceResearch Database (NHIRD)	China	4716	4716
**Engels**	2011	multiorgan	Scientific Registry of Transplant Recipients	U.S.	175732	120654
**Collett**	2010	multiorgan	UK Transplant Registry	UK	37617	25104
**Serraino**	2007	multiorgan	No mention	Italy	2875	1892
**Villeneuve**	2007	multiorgan	Canadian cancer registry	Canada	11155	11 391
**Hoshida**	1997	multiorgan	Multicentre Japan	Japan	2744	1744
**Total**	—	—	—	—	**257206**	**174256**

Table 2 shows summary of the included studies in our analysis with details of the studies. As noted, the 257206 renal transplant recipients were included in this analysis were followed up for a total of 256736 person-years, with a mean follow-up duration of 7. 6125 years (range: 4.8 - 16.0 years). The mean age at transplantation were 44.77 (range: 41.5 - 47.3) years old. Of the 9136 cases that developed cancers and hematological malignancies, this meta-analysis identified 185 cases of liver cancer compared to 84.12 expected cases.

**Table 2:** Demographic details of patients in the included studies

Study	Year	Length of follow-up time	Number of all cancers (n) in kidney transplant cases	Mean follow-up time(years)	Patient-years (years)	Mean age at transplantation (years)	Number of expected cases of liver cancer	Number of identified cases of liver cancers
**Piselli**	2013	1997–2009	395	5.5	39598.0	—	9.40	4
**Cheung**	2012	1972–2011	299	8.2	40 246	47.3	7.92	20
**Krynitz**	2012	1970-2008	2774	5.1	93432	—	—	20
**Li**	2012	1997–2008	320	4.8	22556	41.5	11.80	60
**Engels**	2011	1987–2008	—	—	—	—	44.50	48
**Collett**	2010	1980-2007	4420	16.0	—	—	7.80	19
**Serraino**	2007	—	104	6.5	6931	45.50	—	6
**Villeneuve**	2007	1981–1998	778	7.4	81237	—	2.70	5
**Hoshida**	1997	1970–1995	46	7.4	12982	—	—	3
**Total**	**—**	**—**	**9136**	**7.6125**	**256736**	**44.77**	**84.12**	**185**

### Evidence analysis

**Figure 2:**
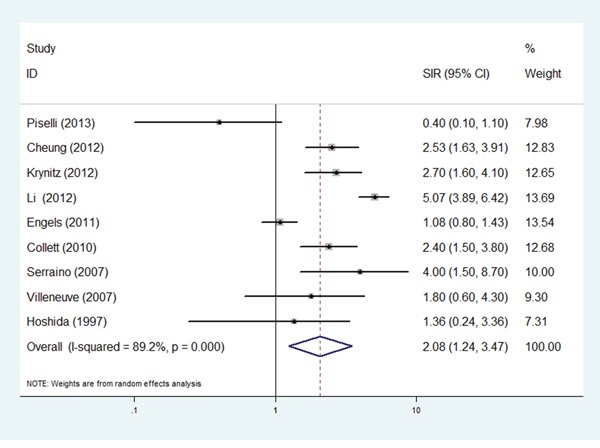
Forest plots of the relative ratios (RRs) and 95% confidence intervals (CIs) for overall risk of liver cancer The squares and horizontal lines correspond to the study-specific RR and 95% CI. The area of the squares reflects the study specific weight.

This meta-analysis for the SIR for liver cancer suggested a significantly increased risk compared to the common population (SIR=2.08, 95%CI: 1.24-3.47; P=0.005; Table 3). Figure 2 shows forest plots for individual and overall RR measures. There was heterogeneity (*I^2^*=89.2%, P _heterogeneity_=0.000) in the pooled analysis.

**Table 3:** Overall SIR and 95% CI in our meta-analysis

Study	Year	SIR	95% Confidence intervals	
			LCI	UCI
**Piselli**	2013	0.40	0.10	1.10
**Cheung**	2012	2.53	1.63	3.91
**Krynitz**	2012	2.70	1.60	4.10
**Li**	2012	5.07	3.89	6.42
**Engels**	2011	1.08	0.80	1.43
**Collett**	2010	2.40	1.50	3.80
**Serraino**	2007	4.00	1.50	8.70
**Villeneuve**	2007	1.80	0.60	4.30
**Hoshida**	1997	1.36	0.24	3.36
**Combined**	**—**	**2.178**	**1.360**	**3.488**

**Figure 3:**
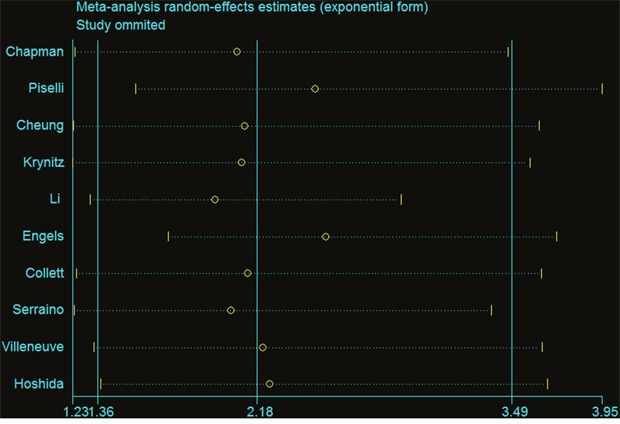
Results of sensitivity analysis liver cancer risk in renal transplant recipients

**Figure 4:**
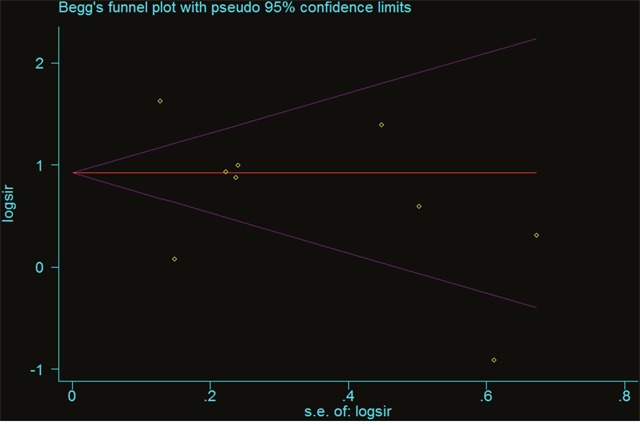
Begg’s funnel plots of liver cancer risk across all related populations

Sensitivity analysis assessed the influence of an individual study on the pooled RR by omitting one study and re-analyzing the results. Our sensitivity analysis indicates the omission of any of the studies led to changes in estimates between 1.844(95%CI=1.209 - 2.814) and 2.428(95%CI=1.579 - 3.733; Table 4). In assessing the association of renal transplantation with the risk of liver cancer risk, we found that no individual altered the significance of the RRs, suggesting the stability and reliability of the overall results (Figure 3). With limited information available, we could not detect any sources contributing to the substantial heterogeneity. Potential publication bias of the studies was assessed using Funnel plots and Begg’s and Egger’s tests. Funnel plot asymmetry was not observed. Publication bias was not evident (t= −0.79, p=0.457) (Figure 4).

**Table 4:** Sensitivity analysis

Study	Year	SIR	95% Confidence intervals	
			LCI	UCI
**Piselli**	2013	2.400	1.439	4.003
**Cheung**	2012	1.997	1.101	3.623
**Krynitz**	2012	1.982	1.101	3.571
**Li**	2012	1.844	1.209	2.814
**Engels**	2011	2.428	1.579	3.733
**Collett**	2010	2.015	1.117	3.635
**Serraino**	2007	1.926	1.111	3.341
**Villeneuve**	2007	2.103	1.215	3.642
**Hoshida**	1997	2.146	1.253	3.675
**Combined**	**—**	**2.077**	**1.244**	**3.471**

## DISCUSSION

Our collected data suggests that the increased risk of developing liver cancer in renal transplantation compared with general population (SIR=2.077, 95% CI= 1.244 - 3.471, P=0.005). Tumors such as NHL and Kaposi’s carcinoma, which are rare in the general population, however, are encountered more frequently in solid organ transplantation recipients [23]. Liver carcinoma prevails around the world, the difference of the prevalence and mortality exists due to the genetic background and regional environmental difference. Among the solid organ transplant recipients (e g: kidney transplantation recipients), its incidence rate also enhanced with the organ transplantation [13, 17, 22]. In this meta-analysis, our collected data provide further evidence that renal transplantation recipients are at more risk of liver carcinoma than the general population.

The exact mechanisms of the oncogenesis in renal transplantation recipients are still unknown. Several possible related factors had been proposed. One hypothesis is that cancers result from related viral infection(hepatitis B virus or hepatitis C virus related) and another is reduced immune surveillance against a variety of non-viral tumor antigens [24]. For a part of renal transplant recipients, they were also chronic B virus carrier at the same time and this figure were similar to the local general population [13]. Organ transplant recipients are vulnerable to viral infection or reactivation of latent infection because the initiation of immunosuppressive agents may promote the unchecked viral replication. In this situation that hepatitis virus infected patients with renal transplant, physicians should check virus quantitative at regular intervals, then take corresponding efforts of anti-virus therapy. At the same time, clinical doctors should attach great importance on immunosuppressive agents treatment period and dosage, in order to prevent hepatitis virus reactivation among patients with viral hepatitis. For the end-stage kidney disease (ESKD) patients, dialysis is one important therapy and it can prolong patients’ media kidney transplant age. A great proportion of patients are usually infected before renal transplantation while using hemodialysis facilities. Previous studies demonstrated that hepatitis C has a wide range of prevalence among renal transplant recipients living in different countries [25, 26]. Some important factors that have an impact on HCV infection in subjects receiving hemodialysis facilities are as follows: race, geographic origin of the recipients [27], type of dialysis [28–30], number of blood transfusion [27, 31], history of organ transplantation [32] and hepatitis B virus co-infection. In Italy, among dialysis patients, the prevalence with HBV and or with HCV is high [33]. Because of renal failure, some dialysis patients need some blood transfusion, the potential reason for this situation is due to the lack of erythropoietin which is mainly produced by the kidney [34], then entire body manifest a series of symptoms of anemia like breathlessness, dizziness, poor appetite and decreased exercise tolerance. Some essays reported that hepatitis C is a major cause of transfusion associated hepatitis and also a leading cause of chronic liver disease in transplant recipients [35, 36]. It is also a cause of chronic hepatitis in approximately 10% of all renal transplant recipients [35]. In order to prevent the occurrence of this phenomenon, regarding to the patients of non-infected hepatitis virus recipients, medical organization should pay much attention to the medical facilities. In addition to hepatitis C virus, hepatitis B virus (HBV) is a well-known oncogenic virus and the main risk for hepatocellular carcinoma development. For hepatitis B virus (HBV) infected patients, occult HBV infection is special infected situation in the human body [37, 38]. Occult hepatitis B infection(OBI) is the presence of hepatitis B virus (HBV) DNA in serum or hepatic tissue without detectable hepatitis B virus surface antigen (HBsAg) in serum [39]. Occult hepatitis B infection may be reactivated, then resulting in acute and severe patterns of hepatitis B virus, which may also occur after transmission of OBI by transfusion or organ transplant. Immunosuppressed and cirrhotic individuals with OBI prone to viral reactivation and hepatocellular transformation [37], so it requires the usage of highly sensitive and specific molecular biology techniques. In the human body, some patients are involved in the immunosuppression situation with acquired immune deficiency such as those with HIV and AIDS (especially HIV popular region). Although the majority of the elevated site-specific cancer risks appear to be driven mostly by immune suppression, linked with oncogenic viral or bacterial infections [40], tumor-promoting effects of the immunosuppressive drugs themselves may also contribute [41]. The long term use of immunosuppressive therapy for the graft is another possible factor promoting liver cancer, as reported for other malignancies [17, 22, 42]. Indeed, immunosuppression resulting from inherited deficiency, AIDS or drugs, has been associated with a higher risk of malignancy than in a immunocompetent population [43]. Immunosuprssive regiments such as azathioprine, prednisolone and cyclosporine have been used commonly in recent decades and carry similar risks for carcinogenicity after kidney transplantation [44]. The exact mechanism for carcinogenicity is unknown. It potentially produces the cytokines that regulate tumor growth, metastasis and angiogenesis. In addition, azathioprine also impair the ability to repair the genetic slicing through acting on the DNA and RNA ways [45]. Other studies have found that the use of mTORi may reduce the overall risk of solid cancers in KT recipients [15, 45]. Among latent infection patients, it is necessary to adjust the dose of immunosuppressant agents according to the patients’ post-operation situation. Some studies also reported other known factors such as age, sex, history of smoking, underlying disease leading to transplantation and history of prior cancers, these known factors maybe influence deeply for the transplant recipients [44, 46]. For instance, if the recipients had a history of alcoholic liver cirrhosis, while in the study without excluding from the transplants, thus increasing the frequency of KT recipients at higher risk of liver cancer.

There are some limitations in this meta-analysis. 1) Despite the funnel plot and Egger’s test showed the absence of publication bias, some important studies except for English publications or unpublished date related to our studies may have been missed, especially when the pooled increased estimate for liver cancer after transplantation was generated from the limited publications. 2) This meta-analysis included several different genetic background, medical situation, life styles, no detailed information for us to perform an adjustment for these potential confounders, such as the period of chronic hepatitis virus carrier, the period of alcoholic liver cirrhosis, obesity and so on. 3) In our collected data, although all studies used the general population as the reference population, the criteria used for matching might be different applied, 4) In our screened studies, two important indices, mean age at diagnosis of malignancy and mean time to develop liver cancer, were not measured in the included studies. 5) The renal transplant recipients were not screened for liver cancer before kidney transplantation (especially chronic hepatitis virus carrier). What’s more, the length of time from transplantation to the diagnosis of the liver cancer was not clear in our results. So we could not exclude the recipients of possible pre-existing tumors in these recipients. 6) Related information on the type and the dosage of immunosuppressive agents were not available for the further subgroup analysis. Some studies demonstrated that low-dose cyclosporine was associated with a lower rate of malignancy than high-dose cyclosporine in RTRs [47].

## MATERIALS AND METHODS

### Literature search

In designing our meta-analysis, a systematic and comprehensive PubMed literature search and Web of Science Databases search updated through September 2015 was conducted to identify studies involving the risk of liver cancer in renal transplantation recipients. The search was restricted to studies published in English and to those including only humans. Search key terms used were ‘renal transplantation’, ‘kidney transplantation’, ‘liver’ and ‘cancer’. The search results were restricted to the presence of the key terms in the title, abstracts and unpublished reports were excluded.

### Inclusion and exclusion criteria

Eligible studies were selected for meta-analysis if they conform to the following inclusion criteria:(1) studies must be population-based cohort studies in renal transplantation recipients; (2) SIR were calculated with 95% confidence intervals in transplant recipients as compared to the general population. (3) Sufficient data on the incidence rate, SIR or relative risk (RR) of liver cancer and enough numbers of patients should be included in the studies. The following exclusion criteria were used: (1) other organ transplantation studies; (2) they were case series, case-control studies, or case reports; (3) they evaluated liver cancer following transplantation of organs other than the kidney; or (4) they evaluated cancers other than liver cancer following renal transplantation.

### Data extraction

The final date from all qualified publication were independently by two investigators (PC and TW) using the inclusion criteria above. They reached an agreement on all items after reducing any form of bias. Important information extracted from each study included: the first author, publication year, the type of transplantation, data source, geographic origin, number of patients, number of renal transplantation cases, length of follow-up time, number of all cancers in kidney transplant cases, mean follow-up time(years), patients-years(years), mean age at transplantation(years), number of expected cases of liver cancer, number of identified cases of liver cancer, and the SIRs of commonly known cancers and liver cancer.

### Statistical analysis

In this meta-analysis, we obtained an estimate from each study of the unadjusted relative risk (RR) with 95% confidence intervals (CIs) to assess the strength of liver cancer in renal transplantation recipients compared to the general population. Because of possible heterogeneity in our studies, a random-effects model was used to pool effects for RR [48]. Heterogeneity between studies, namely differences in study outcomes, was calculated using the chi-squared-based Q-statistic test. The models of analysis for the pooled RRs were based on the P value. By estimating *I^2^*, which was documented for the percentage of the observed between study variability due to heterogeneity rather than chance, with *I^2^*<25%, 25%-75% and >75% representing low, moderate and high degree of inconsistency, respectively.

We assessed the effects of individual study data on the pooled RR through one-way sensitivity analysis. After the sequential removal of each study, the remaining studies could be used to evaluate the stability of the results. At the same time, we assessed publication bias by using a funnel plot and Begg’s test to find out whether there was a bias towards publication of studies [49]. Funnel plot asymmetry was assessed using Egger’s linear regression method on the natural logarithm scale of the RR. A symmetric plot suggested publication bias, with a P value <0.05 indicating significant publication bias. All statistical analyses were performed using the STATE 11.0 software (version 11; STATA Corp, college station, TX, USA).

## CONCLUSIONS

In conclusion, our meta-analysis showed an increased risk of developing liver carcinoma in transplant recipients (especially chronic hepatitis virus carrier). Renal transplant recipients should therefore be screened cautiously for their liver function and adjusted the dosage or period of immunosuppresive agents according to patients’ clinical examination results. If they are the recipients with chronic hepatitis, periodic ultrasound and alpha fetoprotein monitering every 6-12 months are recommended among chronic hepatitis carriers because early detection of tumor can have a higher chance of receiving treatment [50].
